# Plants as silent teachers: bridging plant biology, human physiology, and eastern traditional practices through molecular insights

**DOI:** 10.1080/15592324.2026.2654822

**Published:** 2026-04-02

**Authors:** Anil Sharma, Taj Pratap, Koustuv Dalal

**Affiliations:** a Sri Sri University, Cuttack, India; b Mid Sweden University, Sundsvall, Sweden

**Keywords:** Plant communication, sound vibration, stress physiology, epigenetics, *Arabidopsis thaliana*, yoga, Ayurveda, bioenergetics

## Abstract

Plants lack a centralized nervous system, yet they exhibit sophisticated capacities for environmental perception, communication, stress adaptation, and forms of physiological memory. Increasing experimental evidence indicates that many of the molecular mechanisms underlying these processes—such as reactive oxygen species (ROS) signalling, calcium fluxes (Ca^2+^), nitric oxide (NO) pathways, mitogen-activated protein kinase (MAPK) cascades, and epigenetic regulation—are conserved across biological kingdoms. In this perspective article, we examine how plant systems, particularly *Arabidopsis thaliana*, may serve as experimentally tractable models for investigating conserved stress-related signalling processes that also operate in human physiology. We further review evidence for plant communication through volatile organic compounds, acoustic vibrations, bioelectrical signalling, and mechanical cues, highlighting mechanistic parallels in signal perception and response integration. Building on these shared molecular nodes, we cautiously explore whether plant-based experimental platforms could be used to test environmental variables—such as sound exposure, rhythmic mechanical stimulation, and oxygen-related modulation—that are also relevant to certain contemplative or lifestyle practices. This framework is hypothesis-generating rather than confirmatory and warrants further evaluation. It does not imply equivalence between plant and human experience, but instead proposes that conserved biochemical pathways offer a biologically grounded interface for interdisciplinary investigation. By situating traditional practices within measurable molecular parameters, plant systems may contribute to a more rigorously experimental dialogue among plant biology, stress physiology, and integrative health research.

## 1. Introduction

Modern biomedical science and Eastern knowledge traditions represent distinct yet potentially complementary approaches to understanding life, health, and adaptation. Contemporary emphasizes reductionist, molecular-level explanations and rapid interventions, whereas Eastern systems such as Ayurveda, Yoga, Siddha, and Vedanta emphasize balance, prevention, lifestyle regulation, and subtle energetic processes. Although these traditions have been practiced for millennia, their wider scientific acceptance remains limited, largely due to a lack of mechanistic explanations grounded in molecular biology.

Plants offer a unique bridge between these paradigms. Despite lacking a centralized nervous system, plants display remarkable abilities to sense, integrate, and respond to environmental cues. They communicate with neighboring organisms, discriminate between kin and non-kin, mount systemic stress responses, and retain forms of environmental memory. These functions are mediated by conserved molecular networks that show striking parallels with animal and human systems. Accordingly, plants—particularly genetically tractable species such as *Arabidopsis thaliana*—serve as powerful model systems for exploring universal principles of stress physiology, communication, and adaptation.

Stress-related disorders have become pervasive in modern human societies, prompting renewed interest in contemplative and lifestyle-based interventions rooted in Eastern traditions. Unlike humans, plants do not cognitively interpret stress; instead, they adapt through coordinated molecular, biochemical, and physiological adjustments. Understanding these intrinsic adaptive strategies may inform both sustainable agriculture and human health research. This article aims to (a) synthesize molecular parallels between plant and human stress responses, (b) review mechanisms of plant communication through chemical, acoustic, mechanical, and bioelectrical signals, and (c) propose plants as experimental platforms for evaluating subtle environmental interventions relevant to Eastern practices.

## 2. Evolutionary and molecular foundations of life

Life on Earth emerged approximately 4.6 billion years ago through abiotic chemical processes driven by energy gradients. Early life forms were prokaryotic, heterotrophic, and anaerobic. The evolution of photosynthesis and oxygenic metabolism fundamentally transformed Earth’s atmosphere, enabling the emergence of aerobic metabolism and complex multicellular organisms. At the molecular level, life is governed by the organization of simple chemical constituents into functional and self-regulating systems.

Bioenergetics constitutes a unifying principle across kingdoms. The utilization of glucose, oxygen, and electron transport chains for adenosine triphosphate (ATP) synthesis forms a conserved energetic backbone in plants, animals, and humans. The emergence of eukaryotic cells through endosymbiosis preserved deeply conserved molecular machinery, underscoring the utility of plant systems for investigating fundamental biological processes relevant to human physiology.

## 3. Plants as model systems for human stress biology

The human genome comprises approximately 3.2 billion base pairs encoding ~19,900 protein-coding genes, whereas the *Arabidopsis thaliana* genome contains ~125 Mb encoding over 25,000 genes. Despite divergence more than one billion years ago, many signaling proteins, transcription factors, and regulatory pathways are functionally conserved. Numerous genes associated with human stress responses and disease have identifiable homologs in plants.

In humans, stress responses are primarily mediated by the hypothalamic–pituitary–adrenal (HPA) axis and the sympathetic–adrenomedullary system, leading to the release of glucocorticoids and catecholamines. These processes involve ROS generation, calcium signaling, nitric oxide, and kinase cascades. Similarly, plant stress perception rapidly activates ROS bursts, Ca^2+^ fluxes, nitric oxide signaling, phospholipid turnover, and MAPK pathways. Such parallels suggest that plants can serve as informative models for investigating conserved redox regulation and stress-adaptive signaling.

Oxygen availability represents another point of convergence. Human organs are obligately aerobic, and breath-regulation practices in Yoga emphasize efficient oxygen utilization without inducing hyperoxia. Plants, as primary producers of atmospheric oxygen and regulators of gas exchange, provide experimentally accessible systems in which oxygen-related signaling and metabolism can be precisely manipulated over short life cycles.

## 4. Molecular communication in plants

### 4.1. Chemical signaling

Plants communicate extensively through volatile organic compounds (VOCs), phytohormones, and root exudates. These chemical signals mediate interactions with conspecifics, herbivores, pollinators, and microbial communities. Herbivore-induced volatiles such as methyl jasmonate can prime defenses in neighboring plants or attract natural enemies of pests. Such context-dependent signaling parallels ligand–receptor communication in animal endocrine and paracrine systems.

### 4.2. Acoustic signaling and sound perception

Sound is a mechanical vibration characterized by frequency and amplitude. While traditionally associated with animal communication, accumulating evidence demonstrates that plants perceive and respond to sound vibrations in a frequency-specific manner.^1-3^ Sound exposure influences seed germination, growth, photosynthesis, stomatal conductance, defense responses, secondary metabolite production, and fruit ripening.

Plants also emit sounds, particularly ultrasonic emissions associated with xylem cavitation under drought stress or mechanical injury. At the cellular level, sound-induced responses involve Ca^2+^ signaling, ROS production, ion fluxes, and transcriptional reprogramming, reflecting conserved mechanotransduction mechanisms.

### 4.3. Mechanical and bioelectrical signaling

Mechanical stimuli such as touch, wind, and vibration induce thigmomorphogenic responses that alter plant architecture and gene expression. Concurrently, bioelectrical signals generated by membrane potentials and ion channel activity coordinate tissue- and organ-level organization. Together, these mechanisms form an integrated plant signaling network analogous to neural integration in animals.^4^^,^^5^

## 5. Plant memory and epigenetic regulation

Plants inhabit highly variable environments and must integrate past experiences to optimize future responses. This capacity, often termed plant memory, is mediated by physiological priming, transcriptional reprogramming, and epigenetic modifications. Stress exposure induces changes in DNA methylation, histone modifications, and chromatin accessibility that can persist beyond the initial stimulus.^6-8^

Epigenetic stress memory has been documented in response to drought, temperature extremes, salinity, and pathogen exposure. Comparable mechanisms operate in animals and humans, where environmental factors influence gene expression and long-term health outcomes. Plants therefore, provide accessible systems to investigate the dynamics, persistence, and reversibility of epigenetic memory.^6-8^

## 6. Integration with eastern knowledge systems

Eastern traditions emphasize breath regulation, sound (mantra), ritual practices (e.g., Agnihotra), diet, and lifestyle as modulators of health. For journal audiences, these practices can be discussed as *candidate interventions* that may influence conserved physiological processes—particularly oxygen utilization, autonomic regulation, redox balance, endocrine signaling, inflammation, and stress-responsive gene regulation—without presuming non-operational constructs (e.g., “subtle energies”) as empirical variables. Building a bridge to plant science is feasible because many proximal signaling nodes (e.g., ROS, Ca^2+^, NO, MAPKs, and chromatin remodeling) are shared across kingdoms.

### 6.1. Yoga as a model of breath–movement regulation

Yoga is commonly operationalized in biomedical research as a combination of postures (asanas), breath regulation (prāṇāyāma), and attention/relaxation components. These elements plausibly converge on (a) respiratory and oxygen-related physiology, (b) autonomic balance, and (c) stress-related endocrine and inflammatory pathways. In the present integrative framework, the “time-to-effect” challenge in human studies (heterogeneity in adherence, lifestyle, comorbidities, and exposure history) motivates the use of plants as rapid, controllable systems to test mechanistic hypotheses.^9^^,^^10^

A translationally cautious plant analog is to treat Yoga-relevant components as environmental inputs that can be standardized and measured—e.g., (i) oxygen and carbon dioxide regimes, (ii) rhythmic mechanical stimulation (gentle periodic motion), and (iii) controlled acoustic environments—then quantify conserved molecular readouts (ROS dynamics, Ca^2+^ fluxes, antioxidant enzymes, phytohormone signatures, MAPK activation, and transcriptomic/epigenomic responses). These experiments can be conducted over short plant life cycles and across generations to assess the stability of responses.

### 6.2. Meditation as attentional training with physiological correlates

Meditation practices vary substantially, but a limited clinical and psychophysiological studies frame meditation as attentional training that modulates stress reactivity. While plants do not possess cognition, plant systems can still support mechanistic exploration by focusing on downstream, conserved physiology that meditation is hypothesized to affect in humans (e.g., reduced stress reactivity and altered redox/inflammatory balance). In plants, comparable outcomes can be operationalized as altered stress sensitivity (drought/heat/salinity assays), changes in ROS homeostasis, and modified hormonal and transcriptional responses under standardized stress exposure.^11^^,^^12^

Accordingly, the plant model's contribution is not to claim “meditation-like” states in plants, but to provide a rigorous platform for testing whether environmental correlates associated with meditative practices (e.g., reduced noise, stable light–dark cycles, specific soundscapes) produce reproducible molecular signatures and phenotypic resilience.

### 6.3. Sudarshan Kriya as rhythmic breathing with testable molecular hypotheses

Sudarshan Kriya (SK) is a structured rhythmic breathing practice that, in human studies, is frequently discussed in relation to stress reduction and improved well-being. The key feature for mechanistic translation is rhythmic modulation of breathing, which changes gas exchange and may influence autonomic and redox-related processes. For plant-based experimental design, SK-inspired hypotheses can be translated into controlled manipulations of gaseous environment (O₂/CO₂ dynamics), humidity, and rhythmic mechanical/acoustic cues.

A practical experimental roadmap is to use Arabidopsis thaliana (and at least one crop species for external validity) to test whether SK-analog interventions are effective.^13-15^

- Reprogram stress signaling (ROS–Ca^2+^–NO–MAPK modules) under acute and chronic stress.
- Alter defense and metabolic pathways (e.g., phenylpropanoid/flavonoid biosynthesis) associated with antioxidant capacity.
- Induce priming or memory detectable as enhanced tolerance upon re-stress and/or stable epigenetic marks.
- Show frequency specificity when sound/vibration is used as a component of the intervention.

These plant experiments can be paired with a conservative translational interpretation: plant findings identify candidate pathways and measurable biomarkers that can be prioritized in subsequent human studies, rather than serving as direct evidence of equivalence between plant and human experiences.

### 6.4. Agnihotra and mantra as environmental inputs

Agnihotra and mantra are traditionally framed as ritual and sound-based practices. In a scientific context, they can be treated as complex environmental inputs with potentially separable components: (a) acoustic stimulation (frequency/intensity patterns), (b) thermal/particulate/aerosol exposure from combustion, and (c) contextual timing and environmental conditions. Plants offer a tractable system to test whether these inputs yield consistent changes in growth, pathogen resistance, VOC emissions, metabolite profiles, or microbial community structure.

The central working principle for operationalization involves specifying measurable inputs and outcomes, using appropriate controls (silent/no-sound, heat-only, aerosol-only, randomized sound), and pre-registering primary endpoints. This approach supports respectful engagement with traditional practices while meeting scientific standards for causality and reproducibility.

## 7. Future research directions

Future studies should identify conserved molecular signatures elicited by sound, mechanical stimulation, and oxygen-related interventions across plant and human systems. Integrative transcriptomic, proteomic, metabolomic, and epigenomic approaches in plant models can provide rapid insights into mechanisms underlying subtle environmental influences. Therefore, extensive literature review, empirical experiemnets and further evaluations are highly warranted. Such work may accelerate the development of evidence-based holistic interventions for agriculture and medicine.

## 8. Conclusion

Plants demonstrate that complex adaptive behavior can emerge from decentralized yet highly coordinated molecular networks. Their capacity to integrate environmental signals through ROS dynamics, calcium waves, electrophysiological signaling, hormonal regulation, and chromatin remodeling underscores the sophistication of non-neural biological systems. These conserved signaling architectures provide experimentally tractable platforms for studying how organisms perceive and respond to environmental variability.

The purpose of this integrative framework is not to equate plant physiology with human subjective experience, but to identify shared biochemical nodes that may support hypothesis-driven cross-disciplinary research. By focusing on measurable molecular processes rather than metaphorical parallels, plant systems can be positioned as controlled experimental models for testing how defined environmental inputs influence stress-related signaling pathways.

Whether such findings ultimately inform translational applications in human physiology remains an empirical question. Nonetheless, plant models offer practical advantages—genetic tractability, short life cycles, and ethical accessibility—that allow rapid dissection of stimulus-specific signaling and epigenetic adaptation. Careful mechanistic work in plant systems may therefore contribute to a more rigorous and biologically grounded dialog between plant science, stress biology, and integrative health research.

Future progress will depend on clearly defined experimental designs, evaluations, cautious interpretation of cross-kingdom comparisons, and a sustained commitment to molecular precision. By maintaining these boundaries, plant research can continue to illuminate fundamental principles of environmental signal integration and adaptive resilience across living systems.
